# Newly synthesized peripherally octa-substituted zinc phthalocyanines carrying halogen terminated phenoxy-phenoxy moiety: comparative photochemical and photophysical features

**DOI:** 10.3906/kim-2007-56

**Published:** 2020-12-16

**Authors:** Erkan KIRBAÇ, Ali ERDOĞMUŞ

**Affiliations:** 1 Department of Chemistry, Yıldız Technical University, İstanbul Turkey

**Keywords:** Photochemistry, photophysics, zinc phthalocyanine, octa-substituted, halogen substitution

## Abstract

This study reports the 3 new phthalonitrile derivatives, namely 4, 5 Bis-[4-(4-bromophenoxy) phenoxy] phthalonitrile ( **1** ), 4,5 Bis-[4-(4-chlorophenoxy) phenoxy]phthalonitrile ( **2** ), and 4, 5 Bis[4-(4-fluorophenoxy) phenoxy] phthalonitrile ( **3** ). Their octa-substituted zinc phthalocyanines ( **4** , **5** , **6** ) are reported for the first time in this study. The resulting compounds were characterized by utilizing some spectroscopic methods, such as UV-Vis, 1HNMR, FT-IR spectroscopy, as well as mass spectraand elemental analysis. To show photosynthesizer’s potential, emission (F **F** ), singlet oxygen (1O2), and photodegradation quantum yields (F∆, Fd) of octa-peripherally phthalocyanines (Pcs) were performed in the solutions, such as biocompatible solvent DMSO (dimethyl sulfoxide) as well as DMF (dimethylformamide) and THF (tetrahydrofuran). Solvent and octa-peripherally binding effect of the halogen (Br, Cl, F) terminated phenoxy-phenoxy groups on phthalocyanine rings for photophysicochemical properties ( **4** , **5** , and **6** ) were compared with the tetra-peripherally and tetra nonperipherally substituted derivatives. The new dyes ( **4** to **6** ) may be evaluated in photodynamic therapy (PDT) of cancer as photosensitizers due to efficient 1O2 from 0.55 to 0.75.

## 1. Introduction

Phthalocyanines (Pcs) are known as macrocyclic compounds with different blue-green colors and unique spectroscopic properties. After being discovered in 1927 accidentally, Pcs have since found use in dyes [1,2], catalysis [3–5], optical-based electronics [6,7], electrosensing [8], photovoltaic cells [9,10] and even medicine, such as PDT[11,12]. All of these uses of Pcs stem from their extended 18-π electron system, which contributes to the important chemical and physical properties of phthalocyanines, and also plays an important role in their theoretical or experimental work [13].

A Pc can be modiﬁed by one or a combination of three methods; these all maintain the core atomic conﬁguration of the central Pc, which are metalation, axial substitution, and tetra- or octa-peripherally or nonperipherally substitution [14]. These binding types can give differentproperties to various applications, such as increasing the solubility of the phthalocyanine molecule and the design of the target molecules with the desired properties.

The most versatile method of modifying a Pc’s properties comes from the Pc’s 16 diﬀerent perimeter substitution points (α and β), as these allow the addition of substituents of almost any composition, electron aﬃnity, polarity, and size. These substituents are what allow Pcs to perform the host of functions that they are used for in the modern industry [15]. Substituents that carry properties aﬀecting the electron distribution of the Pc can, however, aﬀect the Pc’s photophysical properties and are position sensitive [16].

In photodynamic therapy, phthalocyanines are used as second generation photosensitizer agents. Phthalocyanines bind to the amine groups of the antibody selected in accordance with the cancerous cell. When photosensitizer-bound antibody is delivered to the body, it only accumulates in diseased tumor cells without spreading throughout the body. When one of the electrons of the oxygen molecule receives energy from outside, it switches to a different orbital opposite to its direction of rotation and singlet oxygen (1O2) forms. Photophysical and photochemical properties are very important studies to determine the potential of photosensitizer candidates, such as phthalocyanine, to be used in photodynamic therapy. Phthalocyanines are second generation compounds as photosensitizers that have the potential to be used in the treatment of cancer by PDT owing to their appreciate wavelength absorption and the ability to form singlet oxygen effectively [17–19].

The properties of phthalocyanine compounds can be enriched with different substituents. Selected groups can be connected to the tetra-peripherally, tetra-nonperipherally or octa-peripherally or nonperipherally substituted positions at the Pc ring, and the desired photophysical and photochemical properties can be adjusted. The Pcs with the halogen atoms terminated phenoxy-phenoxymoiety at the octa-peripherally substituted were not performed before. Our recent articles show that synthesis, and photochemical and emission properties of tetra-substituted Zn(II) complexes bearing identical groups at nonperipherally and peripherally positions were discussed [20,21]. Octa-substituted phthalocyanines have been reported to have better solubility and lower aggregation tendency [22,23].The goal of the study was to inspect the photosensitizer features of peripherally octa-binding versus tetra nonperipheral and tetra-peripheral positions for zinc phthalocyanine analogs.

## 2. Experimental design

All information about the used materials, equipment, synthesis, emission properties as photophysical and 1O2 efficiency and photostability properties as photochemically are shown in the “Supplementary Information”.

## 3. Results and discussion

### 3.1. Syntheses and characterization

The chemical synthesis routes to new octa-substituted zinc phthalocyanines (4 to 6) are represented in Scheme 1. The Pcs were obtained by the cyclotetramerization of the nitriles (1, 2, and 3) with dryzinc acetate in the presence of DBU catalyst in n-hexanol at reflux temperature under argon atmosphere.

All the compounds were purified by column chromatography after thin layer chromatography studies. Their characterization were performed by using FTIR, 1H NMR and UV-Vis spectroscopic techniques, together with mass spectra and elemental analysis.

Very characteristic FTIR vibrations of C=N triple bond were monitored at 2233 (for 1), 2224 (for 2), and 2226 (for 3) cm–1 for the phthalonitriles. The vibration of ether bonds (C-O-C) for the nitriles were observed at 1240 cm–1 (1), 1205cm–1(2), and 1250 cm–1(3), respectively. Aromatic C-H bond vibration peaks occurred at around 2970–3094 cm–1 for all the new nitriles. The 1H NMR spectrum of the nitriles (1 to 3) gave for aromatic protons signals with δ between 7.90 and 6.94 (for 1), 7.28and 6.90 (for 2), and 7.19 and 7.04 (for 3), integrating for a total of 18 protons, respectively. The mass value of the phthalonitriles was obtained by the gas chromatography-mass (GC-MS) technique for 1, and time-of-flight mass spectrometry (TOF-MS) technique for 2 and 3. The molecular ion peaks were signed at m/z 654 for 1, 587 for 2,and 555 for 3.

To achieve peripherally octa-substituted Zn(II)Pcs from their precursors, the template effect of Zn(OAc)2was applied as central ion effect of Zn(II). Then cyclotetramerization of the nitriles, the distinctive carbon-nitrogen triple bond (C=N) vibration signals disappeared on the FTIR spectra of Pc complexes, the disappearing the peaks, the evidence of the made up of Pcs. The C-O-C vibrations were observed at 1232, 1187 cm–1for 4, 1203, 1186 cm–1for 5, and 1247, 1185 cm–1for 6, respectively. Aromatic carbon hydrogen single bond (CH) peaks occurred at 3107 (4), 3041 (5), and 3070 cm–1 (6) for phthalocyanines.

The purity of octa-substituted Zn(II)Pc derivatives were also checked by 1H NMR with both of the groups, and Pc skeleton protons appeared in their respective regions. In the 1H NMR spectrum of 4 to 6, the aromatic Pc and substituent aromatic protons appeared between 7.60–6.55 ppm for 4,7.65–6.90 ppm for 5, and 7.20–6.71 ppm for 6. In the MS of peripherally octa-substituted Zn(II)Pcs, molecular (M) ion peak was observed at m/z 2682 [M]+ (for 4), (M+H) ion peaks were seen 2328 [M+H]+ and 2196 [M+H]+ for 5 and 6, respectively (Figure S1), as confirmed the proposed structures. Elemental analysis data also supported the proposed chemical formulas for the precursors and their Pcs (1 to 6) as can be seen in the experimental part.

**Figure 1:**
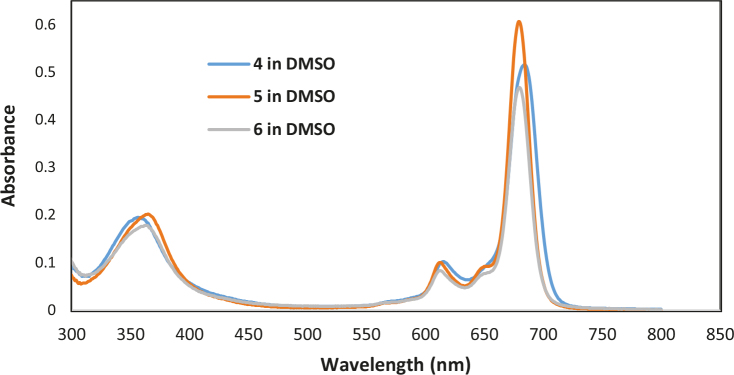
UV-Vis absorption spectra of 4, 5, and 6 in DMSO concentration at 2 × 10–6 moldm–3.

The electronic ground state spectra of Zn(II)Pcs (4 to 6) were performed inTHF, DMF, and DMSO at room temperature (An example for 4, 5, and 6 in DMSO is given in Figure 1). The Q-bands of compounds (4 to 6) appeared at 681, 677, and 676 nm in DMF; 684, 680, and 680 nm in DMSO; and 678, 675, and 675 in THF, respectively (Table 1). Their B-bands were seen between 340 and 365 nm for all the compounds. The logarithmic molar absorption coefficient values of the bands are listed in Table 1. Additionally, the vibrionic band peaks of4, 5 and 6 were recorded between 609 and 615 nm assigned to n - π* transitions for the complexes. Generally, the larger metal ions cause more red-shift of the Q-band. Axial ligandation of metal ions also makes the Q-band shift to the red region. Moreover, Q-bands of the nonperipherally substituted [21] Pcs show up a bathochromic shift in comparison to those of their tetra- and octa-peripheral analogs. To conclude, the Q-bands of the MPcs (4 to 6) shift to the red region in the following order: 5 ≤  6 < 4 in the solvents used. When compared to the previous tetra-peripheral and nonperipheral analogs [20,21] in DMSO, THF, and DMF, the Q-band values of peripherally octa-binding ones are blue-shifted relative to those of nonperipherally derivatives, but they have almost the same value as tetra-peripherally patterns. Type of halogen atoms (F, Cl, and Br) on the phenoxy-phenoxy substituent did not show a crucial change in the Q bands maximum for the Pc rings.

**Table 1:** Spectral parameters of 4, 5, and 6 in DMF, DMSO, and THF.

Comp.	Solvent	Q-band	(log ε)	Excitation	Emission	Stokes shift
		ƛmax, (nm)		ƛEx, (nm)	ƛEm, (nm)	ΔStokes, (nm)
4	DMF	678	5.39	679	689	11
	DMSO	684	5.39	682	689	5
	THF	675	5.27	673	686	11
5	DMF	678	5.14	676	691	13
	DMSO	680	5.16	681	690	10
	THF	675	5.17	676	684	9
6	DMF	678	5.39	690	690	12
	DMSO	680	5.33	692	692	12
	THF	675	5.42	687	687	12

**Figure 2:**
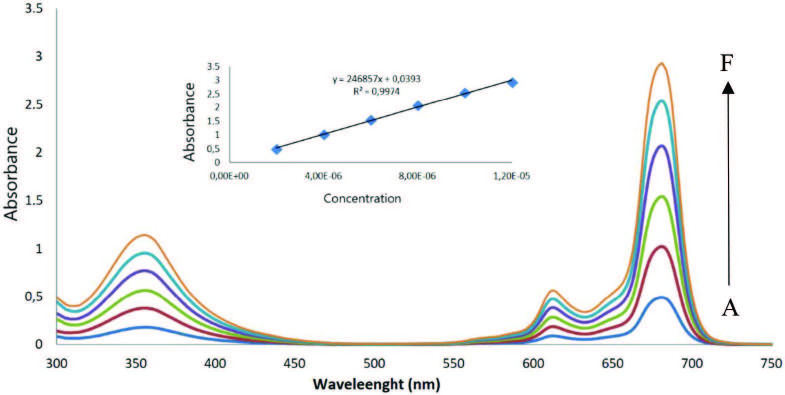
Absorption spectra of 4 in DMF at different concentration: 2 × 10–6 (A), 4 × 10–6 (B), 6 × 10–6 (C), 8 × 10–6 (D), 10 × 10–6 (E), 12 × 10–6 (F) moldm-3.

Aggregation behavior reduces the solubility of the Pcs in various solvents and subsequently weakens their performance in a wide range of scientific and technological fields requiring high soluble materials. Therefore, it matters to recognize and improve the factors affecting the aggregation behavior of Pcs. Change in concentration of Pcs, the solvent nature, and the temperature can alter aggregation as well as the size and position of the substituent. Spectral properties of Pcs as a function of the electronic states change by enhancement of π-stacking which derange of the electronic states. Furthermore, the study of the electronic absorption spectra of Pcs is a useful approach for the measurement and management of the aggregation [24,25]. The concentrationeffect on aggregation properties of compounds 4, 5, and 6 was examined in different molarity of THF, DMSO, and DMF,ranging from 2 × 10–6 to 12 × 10–5 M. As concentration increased, the absorbance enhanced directly in a constant value, and no new band was observed. Since all compounds obey the Lambert-Beer law, aggregation does not rely on concentration at the studied range of concentration (Figures 2 and S2 were given for 4 in DMF). 

## 4. Photophysical and photochemical properties

### 4.1. Emission spectra and fluorescence quantum yields

**Figure 3:**
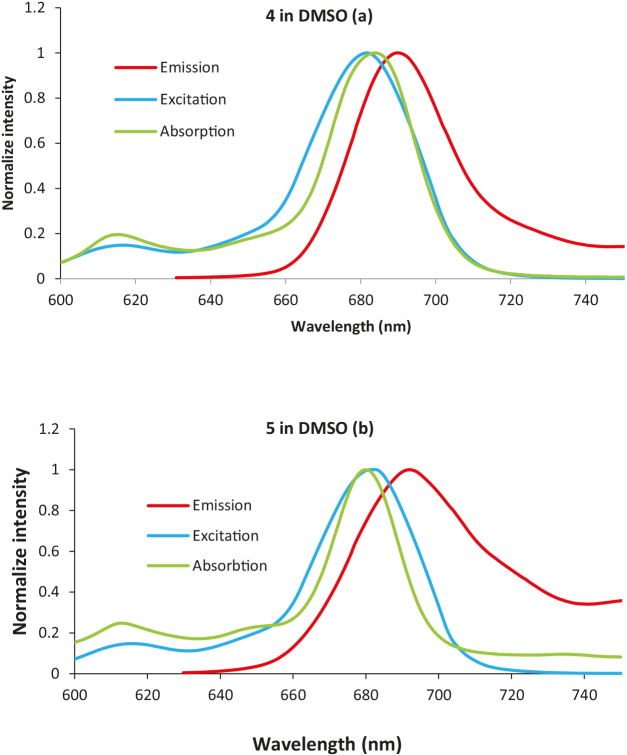
Absorption, excitation, and emission spectra of the compounds 4 (a) and 5 (b) in DMSO.

The emission properties of photosensitizing molecules are important measures for the evaluation of their application as biological imaging agents. Among a vast range of materials, Pcs include specific chemical and physical features that make them appropriate compounds in this respect. Therefore, the spectrophotometric and spectrofluorometric properties of Pcs are studied to determine the suitability of these molecules as biological imaging materials [26]. Fluorescence features of the complexes (4 to 6) were researched into in DMSO, THF,and DMF. The emission, excitation, and ground state spectra of the macrocyclic molecules (4 and 5) in DMSO are depicted in Figures 3 and S3. Octa-peripherally substituted zinc phthalocyanine derivatives showed similar emission, excitation, and absorbance characteristics with tetra-substituted peripheral and nonperipheral derivatives, except for minor differences in the wavelengths [20,21]. Maximum peak of the emission was seen at the following values: 689 nm for 4, 691 nm for 5, 690 nm for 6 in DMF; 689 nm for 4, 690 nm for 5, 692 nm for 6 in DMSO; and 686 nm for 4, 684 nm for 5, 687 nm for 6 in THF (Table 1), respectively. The excitation spectra were mirror images of the emission spectra for all Pcs.

The effect of substituent nature and solvent type on the values of ΦF were examined, and the highest value was obtained for complex **5** in DMSO(ΦF = 0.13). Generally, nonperipherally substitution leads to lower values of ΦF due to its proximity to the Pc ring, and the octa-substituted Zinc(II)Pc complexes (4, 5, and 6) have lower values of ΦF in comparison to their ZnPc patterns [20,21]. Fluorescence quantum efficiencies of the octa-connected Pcs are higher than unsubstituted ZnPc; ΦF = 0.18in DMSO [21]. Significant effect of halogen atom types (F, Cl, Br) on the Pcs on ΦF values of 4, 5 and 6 were not observed in the solutions. However, the F-substituted derivative showed higher florescence efficiency in all solvents.

### 4.2. Singlet oxygen quantum yields(ΦΔ)

The effective singlet oxygen “1O2” generation is the most important element of photodynamic therapy after combination of light, photosensitizers, and molecular oxygen. Due to the high reactivity of singlet oxygen, some biological macrosystems, such as nucleic acid, proteins, and lipid membranes, can be damaged and finally induce cell death. Energy transfer from photosensitizer to molecular oxygen should be as efficient as possible to obtain more singlet oxygen. This study aims to evaluate their effectiveness for the production of singlet oxygen since phthalocyanines containing phenoxy-phenoxy group with terminated halogen atoms (F, Cl, Br) seem to be suitable for inducing intersystem crossing. The effect of some factors consisting of the substituent, and the terminated halogens atom types on the “1O2” quantum yields were investigated by applying a photochemical method based on the chemical quenching. The measurements “1O2” yield are studied in the 3 solvents (DMF, THF, and DMSO) to determine whether the new Pcs were advisable for photodynamic therapy application. u1d51ure 4 shows absorbance changes of DPBF observed during photolysis of zinc phthalocyanine complexes 4, 5, and 6 in DMSO by using UV-Vis spectroscopy. The degradation rate of DPBF is related to singlet oxygen production. No change in the Q band maxima of the Pcs was observed during the ΦΔ determinations, which confirms that the sensitizers are not disrupted by 1O2 attack (Figure S4) [26]. The ΦΔ values are for 4 (0.69), 5 ( 0.61), 6 (0.67) in DMF; for 4 ( 0.60), 5 ( 0.55), 6 (0.67) in DMSO; for 4 (0.68), 5 (0.75), and 6 (0.73) in THF. The ΦΔ values of 3 phthalocyanines were generally bigger in THF than DMF and DMSO. Chosen moiety on Pc skeleton increased the generation of 1O2 compared to unsubstituted zinc phthalocyanine in DMF and THF (Table 1). When the effect of the halogen atom types was examined, there was no important difference depending on the halogen atom type. However, those with an F-end showed a higher singlet oxygen yield in DMSO, those with a Cl-end in THF, and those with a Br-end in DMSO. Octa-substituted zinc phthalocyanines (4 to 6) showed almost the same 1O2 quantum yields compared to the previously obtained tetra-substituted peripherally and nonperipherally Zn(II)Pcs analogs bearing the same groups [20,21].

**Figure 4:**
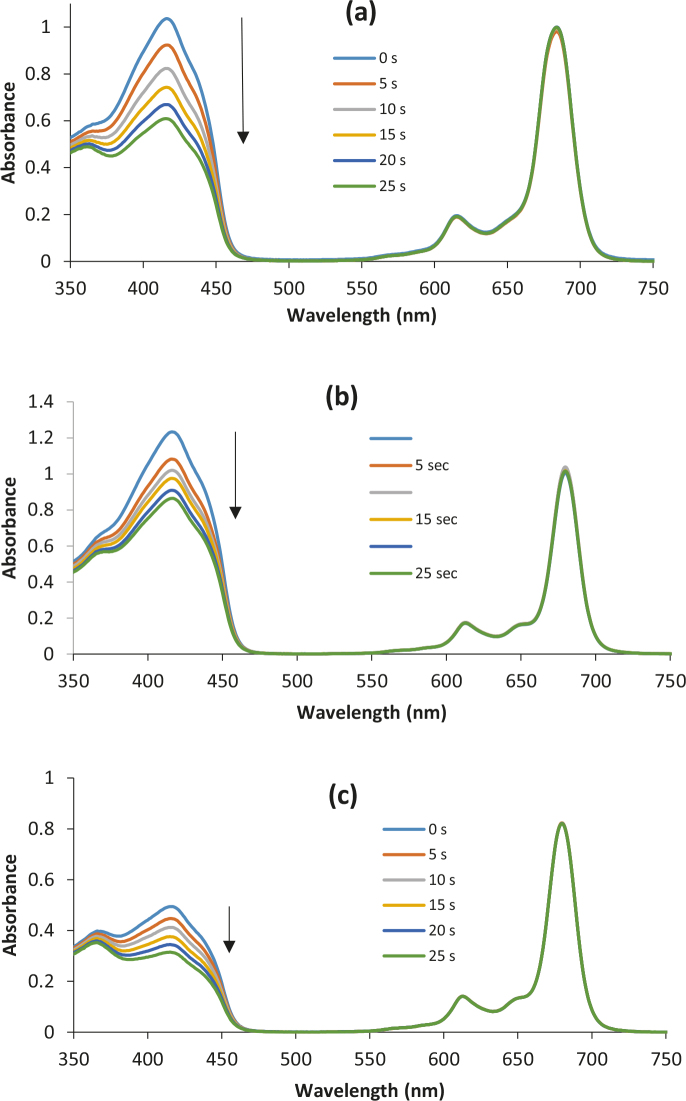
A typical spectrum for the determination of singlet oxygen quantum yield of for complex 4 (a), 5 (b), 6 (c) in DMSO at a concentration 6 × 10–6 moldm–3.The light intensity of 7.05 × 1015 photons s–1 cm–2 was used for FDwith 5 s intervals.

### 4.3. Photodegradation quantum yields under the light (Φd)

**Figure 5:**
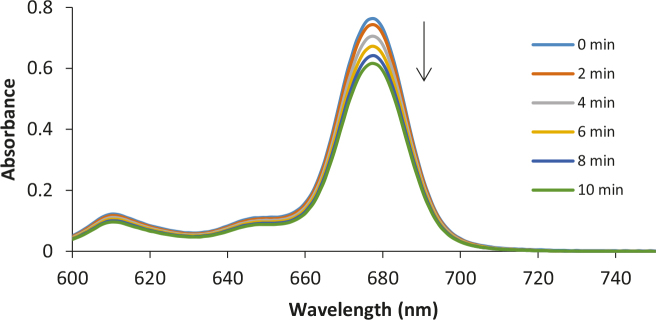
A typical spectrum for the determination of photodegradation of complex 6 in DMF.

The effective photosensitizers during the photodynamic therapy applications should be stable under the applied light. This stability is necessary to maintain the efficiency of the photosensitizer molecule in terms of singlet oxygen production and to keep the drug concentration unchanged. Photodegradation is the oxidative degradation to determine the stability of a compound under photo irradiation applied and can be defined by photodegradation quantum yield. These processes were performed in THF, DMSO, and DMF by examining the falling away in the intensity of the maximum Q band of the complexes by the time. The photodegradation quantum yields are shown in Table 2. The obtained results show that synthesized complexes are stable to photochemical degradation and are much more resistant, especially compared to unsubstituted ZnPc. To measure Φd value, the absorbance (Q-band maxima) changes observed for 6 in DMF are shown in Figures 5 and S5. Highly stable phthalocyanine molecules give values of Φd as low as 10−6, and for unstable Pcs values of Φd the order 10−3 have been reported [27]. The order of photochemical stabilities of the compounds were 4 > 5 > 6 in DMSO, and 6 > 4 > 5 in DMF, respectively. Φd of 4, 5, and 6 samples displayed high photostability under a light intensity of 2.50 × 1016 photons s-1 cm-2 (u1d54). Not all of the complexes showed important photodegradation in measurements taken in THF. The complexes were highly stable in THF, while they showed the highest photochemical instability in DMF.

**Table 2:** Photophysical and photochemical properties of 4, 5, and 6 in DMF, DMSO, and THF.

Compound	Solvent	FF	Fd (10-4)	F∆
4	DMF	0.07	11.0	0.69
	DMSO	0.03	0.40	0.60
	THF	0.10	----	0.68
5	DMF	0.08	15.0	0.61
	DMSO	0.13	1.00	0.55
	THF	0.10	-----	0.75
6	DMF	0.10	8.0	0.67
	DMSO	0,11	6.0	0.67
	THF	0.09	------	0.73

## 5. Conclusion

In this study, a new series of zinc phthalocyanine compounds carrying F (6), Cl (5), and Br (4) halogens terminated phenoxy-phenoxy moiety to octa-substituted position were successfully synthesized. Structural characterization of the resulting compounds (1 to 6) was performed using a number of diverse spectroscopic approaches. All data matched the proposed structures. Aggregation behaviors of the zinc Pcs were carried out at increasing molarity in THF, DMF, and DMSO. Additionally, the effect of solvent nature on the aggregation behavior of the zinc Pc was examined. As concentration increased, the absorbance enhanced directly in a constant value, and no new band was observed. Thus, nonaggregated behavior of the Pcs suggest that PDT applications are useful in the solutions. When the effect of the solvent on singlet oxygen production was examined, admirable photophysicochemical results were obtained among the 3 solvents used. Photophysical and photochemical properties of zinc complexes bearing the same substituent terminated different halogens (F, Cl, and Br) at octa-peripherally positions were also studied comparatively to their tetra-peripherally and nonperipherally patterns. Important increases in the 1O2 quantum yields were realized in the presence of the selected group and diamagnetic zinc atom as the central atom. Compared to the type of halogen atoms via phenoxy-phenoxy groups improve photophysicochemical properties. The results of photochemical measurements show that the complexes have suitable photodegradation stability with applicative “1O2” efficiencies ranging from 0.55 to 0.75.

Supplementary Materials
