# Supplementary material for: Newly synthesized peripherally octa-substituted zinc phthalocyanines carrying halogen terminated phenoxy-phenoxy moiety: comparative photochemical and photophysical features
Source: Turk J Chem. 2020 Dec 16;44(6):1556–64. doi: 10.3906/kim-2007-56 (PMC7765766; doi:10.3906/kim-2007-56)
Supplement: Supplementary file 1 — Supplementary Materials [file turkjchem-44-1556-sup001.pdf]

SUPPLEMENTARY INFORMATION

Newly synthesized peripherally octa-substituted zinc phthalocyanines carrying halogen terminated phenoxy-phenoxy moiety; comparatively photochemical and photophysical features

Erkan KIRBAÇ, Ali ERDOĞMUŞ*

Department of Chemistry, Yildiz Technical University, 34210 Esenler, Istanbul-Turkey

* Corresponding author. Fax: +90 264 295 74 26. E-mail address: aerdog@yildiz.edu.tr

1. Materials and equipment

Dimethylsulfoxide (DMSO), 1-pentanol, methanol, n-hexane, chloroform ($CHCl_3$), tetrahydrofuran (THF), acetone, K_2CO_3, ethanol, and dimethylformamide (DMF) were purchased from Merck, 1,8-diazabicyclo[5.4.0]undec-7-ene (DBU), 1,3-diphenylisobenzofuran (DPBF), 4,5-dicholorophthalonitrile, zinc acetate, zinc phthalocyanine, 4- (4-bromophenoxy)phenol, 4-(4-clourophenoxy)phenol and 4-(4-Flourophenoxy)phenol were purchased from Sigma Aldrich. Column chromatography was performed on silica gel 60 (0.04–0.063mm).

FT-IR spectra (KBr pellets) were measured with a Perkin Elmer Spectrum One Spectrometer. Absorption spectra in the UV-Visible region were obtained with a Shimadzu 2001 UV spectrophotometer. Elemental analyses were recorded with a Thermo Flash EA 1112 Series.

Fluorescence spectra were done using a Varian Eclipse spectrofluoremeter using 1 cm pathlength cuvettes at room temperature. ^1H NMR spectra were recorded in $CDCl_3$ solutions on a Varian 500 MHz spectrometer.

Photo-irradiations were done using a General Electric quartz line lamp (300W). A 600 nm glass cut off filter (Schott) and a water filter was used to filter off ultraviolet and infrared radiations respectively. An interference filter (Intor, 700 nm with a bandwidth of 40 nm) was additionally placed in the light path before the sample. Light intensities were measured with a POWER MAX5100 (Molelectron detector incorporated) power meter. The mass spectra were acquired on a Bruker Daltonics (Bremen, Germany) MicroTOF mass spectrometer equipped with an electronspray ionization (ESI) source. The instrument was operated in positive ion mode using a m/z range of 50–3000. The capillary voltage of the ion source was set at 6000 V and the capillary exit at 190 V. The nebulizer gas flow was 1 bar and drying gas flow 8 mL/min.

2. Photophysical and Photochemical Studies

2.1. Fluorescence quantum yields

Fluorescence quantum yields (Φ_F) were determined by the comparative method (Eq. 1) [S1],

$$\Phi_F = \Phi_{F(Std)} \frac{F \,.\, A_{Std} \,.\, n^2}{F_{Std} \,.\, A \,.\, n^2_{Std}} \qquad (1)$$

where F and F_{Std} are the areas under the fluorescence emission curves of the samples (**4** to **6**) and the standard, respectively. A and A_{Std} are the respective absorbances of the samples and standard at the excitation wavelengths, respectively. n^2 and n^2_{Std} are the refractive indices of solvents used for the sample and standard, respectively. Unsubstituted ZnPc (in DMSO) (Φ_F = 0.20) [S2], (in DMF) (Φ_F = 0.17) [S3], (in THF) (Φ_F = 0.25) [S4] was employed as the standard. Both the samples and standard were excited at the same wavelength. The absorbance of the solutions at the excitation wavelength ranged between 0.04 and 0.05.

2.2. Singlet oxygen quantum yields

Singlet oxygen quantum yield (Φ_Δ) determinations were carried out using the experimental set-up described in the literature [S5-S8]. Quantum yields of singlet oxygen photogeneration were determined in air (no oxygen bubbled) using the relative method with ZnPc as reference and DPBF as chemical quencher for singlet oxygen, using equation 2

$$\Phi_\Delta = \Phi_\Delta^{Std} \frac{R \,.\, I_{abs}^{Std}}{R^{Std} \,.\, I_{abs}} \qquad (2)$$

where Φ_Δ^{Std} is the singlet oxygen quantum yields for the standard ZnPc (Φ_Δ^{Std} = 0.67 in DMSO [S8] and 0.56 for ZnPc in DMF [S9], and 0.53 for ZnPc in THF [S10] R and R_{Std} are the DPBF photobleaching rates in the presence of the respective samples (**4**, **5** and **6**) and standard, respectively. I_{abs} and I_{abs}^{Std} are the rates of light absorption by the samples (**4**, **5** and **6**) and standard, respectively. To avoid chain reactions induced by DPBF in the presence of singlet oxygen [S9], the concentration of quencher (DPBF) was lowered to ~3 x 10^{-5} mol dm^{-3} Solutions of sensitizer (containing DPBF) were prepared in the dark and irradiated in the Q band region using the setup described above. DPBF degradation at 417 nm was monitored. The light intensity of 7.05 x 10^{15} photons s^{-1} cm^{-2} was used for $\Phi_\Delta$determinations.

2.3. Photodegradation quantum yields

Photodegradation quantum yield (Φ_d) determinations were carried out using the experimental set-up described in the literature [S6-S7]. Photodegradation quantum yields were determined using formula 3,

$$\Phi_d = \frac{(C_0 - C_t) \,.\, V \,.\, N_A}{I_{abs} \,.\, S \,.\, t} \qquad (3)$$

where “C_0” and “C_t” are the sample (**4**, **5** and **6**) concentrations before and after irradiation respectively, “V” is the reaction volume, “N_A” the Avogadro’s constant, “S” the irradiated cell area and “t” the irradiation time, “I_{abs}” is the overlap integral of the radiation source light

intensity and the absorption of the samples (**4**, **5** and **6**). A light intensity of $2.50x10^{16}$ photons s^{-1} cm^{-2} was employed for Φ_d determinations.

3. Synthesis

3.1. 4,5 Bis-[4-(4-bromophenoxy)phenoxy]phthalonitrile (1)

The 4,5-dicholorophthalonitrile (0.39 g 1.97 mmol) was dissolved in dry DMF (10 ml) under inert argon atmosphere and 4-4(bromophenoxy) phenol (1.00 g 3.77 mmol) was added. After stirring for 30 min at room temperature, finely ground anhydrous potassium carbonate (2.0 g 14.47 mmol) was added in portions during two hours with efficient stirring. The reaction mixture was stirred under argon atmosphere at room temperature for 24 h. Then the mixture was dumped into 100 ml could water, and the precipitate was filtered off, and crystallized in methanol and then dried. Finally, the pure powder was dried in a vacuum. Yield: 0.27 g (22%). IR spectrum (cm-1): 2970 (Ar-CH), 2233 (C≡N), 1478 (C=C), 1240, 1095 (C-O-C), 824 (C-Br). ^{1}H NMR ($CDCl_3$): δ = 7.88-7.90 (m, 2H, Ar-H), 7.46-7.52 (m, 4H, Ar-H), 7.04-7.12 (m, 10H, Ar-H). 6.94-7.00 (m, 2H, Ar-H). The results of elemental analysis, Calcd for $C_{32}H_{18}Br_2N_2O_4$: C, 58,74; H, 2,77; N, 4,28%; Found: C, 58.70; H, 2.75; N, 4.23%. MS (GC-MS) m/z: Calc. 654.30; Found: 654.0 $[M]^+$.

3.2. 4,5 Bis-[4-(4-chlorophenoxy)phenoxy]phthalonitrile (2)

The synthesis of **2** was similar to that of **1**, except 4- (4-chlorophenoxy) phenol (1.00 g 4.53 mmol) was employed instead of (1). The amounts of the other reagents were: 4,5-dicholorophthalonitrile, 0.45 g (2.28 mmol) and anhydrous potassium carbonate, 2 g (14.47 mmol).

Yied: 0,23 g (17%). IR spectrum (cm^{-1}): 3094 (Ar-CH), 2224 (C≡N), 1586 (C=C), 1205, 1087 (C-O-C), 826 (C-Cl) . ^{1}H NMR ($CDCl_3$): δ = 6.90-6.94 (m, 4H, Ar-H), 6.98 (m, 4H, Ar-H), 6.98 (m, 8H, Ar-H), 7.10 (m, 2H, Ar-H), 7.25-7.28 (m, 4H, Ar-H). The results of elemental

analysis, Calcd for $C_{32}H_{18}Cl_2N_2O_4$: C, 67.98; H, 3.21; N,4.95%; Found: C, 68.04; H, 3.19; N, 4.91%. MS (TOF-MS) m/z: Calc. 564.1; Found: 587 $[M+Na]^+$.

3.3. 4,5 Bis[4-(4-fluorophenoxy) phenoxy]phthalonitrile (3)

The synthesis of **3** was similar to that of **1**, was employed instead of 4- (4-fluorophenoxy)phenol (1.0 g 4.90 mmol) . The amounts of the other reagents were: 4,5-dicholorophthalonitrile, 0.48 g (2.43 mmol) and anhydrous potassium carbonate, 2 g (14.47 mmol). Yied: 0.28 g (21 %). IR spectrum (cm^{-1}): 3095 (Ar-CH), 2226 (C≡N), 1585 (C=C), 1250, 1084 (C-O-C), 843 (C-F); ^1H NMR ($CDCl_3$): δ = 7.04-7.12 (m, 14H, Ar-H), 7.19 (m, 4H, Ar-H). The results of elemental analysis, Calcd for $C_{32}H_{18}FN_2O_4$, The results of elemental analysis, Calcd for $C_{32}H_{18}F_2N_2O_4$, C, 72.18; H, 3.41; N,5.26%; Found: C, 72.23; H, 3.45; N, 5.31%. MS (TOF-MS) m/z: Calc. 532.0; Found: 555 $[M+Na]^+$.

3.4. (3,4)-Octo[(bromophenoxy) phenoxy] phthalocyaninato zinc(II) (4)

A mixture of 4,5 Bis-[4-(4-bromophenoxy)phenoxy]phthalonitrile (**1**) (0.10 g 0.15 mmol), DBU (0.2 ml, 1.33 mmol) and zinc acetate (0.05 g, 0.50 mmol) in n-hexanol (4.0 ml) was refluxed and stirred under argon atmosphere for 12 h. The resulting green suspension was cooled. The crude product was precipitated by addition of n-hexane, collected by centrifuged and washed with hot hexane, ethanol and methanol. The green product was further purified by column chromatography over a silica gel using a mixture of $CHCl_3$: MeOH (100/ 5 v/v) as eluent. Yield: 0.037 g (36%). UV-Vis (DMF): λmax nm (log ε) 681 (5.39), 613 (4.68), 356 (4.98); UV-Vis (DMSO): λmax nm (log ε) 684 (5.39), 615 (4.71), 356 (4.99); (THF): λmax nm (log ε) 678 (5.27), 611 (4.53), 351 (4.84). FT-IR ν_{max}/cm^{-1} (KBr pellet): (3107 (Ar-CH), 1600 (C=C), 1480 (C=N), 1254, 1232, 1187 (C-O-C) ^1H NMR ($CDCl_3$): δ = 6.96-67,90 (44H, m, Ar-H)., The results of elemental analysis, Calcd for $C_{128}H_{72}Br_8N_8O_{16}Zn$: C, 57.31; H, 2.71; N, 4.18%; Found: C, 57.39; H, 2.68; N, 4.25%. MS (MALDI-MS) m/z: Calc: 2682.0; Found: 2682 $[M]^+$.

3.5. (3,4)-Octo [chlorophenoxy] phenoxy phthalocyaninato zinc(II) (5)

Synthesis and purification was as outlined for **4** except 4-5 bis [4-(4-chlorophenoxy) phenoxy] phthalonitrile (0.10 g 0.17 mmol), (**2**) was employed instead of **1**. Amounts of reagents used in DBU (0.2 ml, 1.33 mmol), zinc acetate (0.1 g, 0.50 mmol) in n-hexanol (4.0 ml). Yield: 0.026 g (25 %). UV-Vis (DMF): λ_{max} nm (log ε) 677 (5.14), 611 (4.32), 365 (4.65); UV-Vis (DMSO): λmax nm (log ε) 680 (5.16), 612 (4.38), 364 (4.69); (THF): λ_{max} nm (log ε) 675 (5.17), 609 (4.36), 357 (4.69). FT-IR ν_{max}/cm^{-1} (KBr pellet): 3041 (Ar-CH), 1592 (C=C), 1481 (C=N), 1203, 1186 (C-O-C) (Pc skeletal). ^1H NMR ($CDCl_3$): δ = 7.65-6.90 (44H, m, Ar-H). The results of elemental analysis, Calcd for $C_{128}H_{72}Cl_8N_8O_{16}Zn$: C,66.07; H, 3.12; N,4.82%; Found: C, 66.16; H, 3.16; N, 4.87%. MS (MALDI-MS) m/z: Calc. 2327; Found: 2328 $[M+H]^+$.

3.6. (3,4)-Octo [fluorophenoxy] phenoxy phthalocyaninato zinc(II) (6)

Synthesis and purification was as outlined for **4** except 4,5 Bis[4-(4-fluorophenoxy) phenoxy]phthalonitrile (0.1 g 0.18 mmol), (**3**) was employed instead of **1**. The amounts of the reagents employed were: DBU (0.20 ml, 1.33 mmol), zinc acetate (0.01 g, 0.50 mmol) in n-hexanol (4 ml). Yield: 0.032 g (31 %). UV-Vis (DMF): λ_{max} nm (log ε) 676 (5.39), 610 (4.64), 362 (4.97). UV-Vis (DMSO): λ_{max} nm (log ε) 680 (5.33), 613 (4.60), 365 (4.89); (THF): λ_{max} nm (log ε) 675 (5.42), 609 (4.67), 356 (5.00).

FT-IR ν_{max}/cm^{-1} (KBr pellet): 3070 (Ar-CH), 1605 (C=C), 1486 (C=N), 1247, 1185 (C-O-C) (Pc skeletal) ^1H-NMR ($CDCl_3$): δ = 7.20-6.70 (44H, m, Ar-H). The results of elemental analysis Calc. for $C_{128}H_{72}F_8N_8O_{16}Zn$: C, 70.03; H, 3.31; N, 5.10%; Found: C, 70.11; H, 3.35; N, 5.16%. MS (MALDI-MS) m/z: Calc. 2195.0; Found: 2196 $[M+H]^+$.

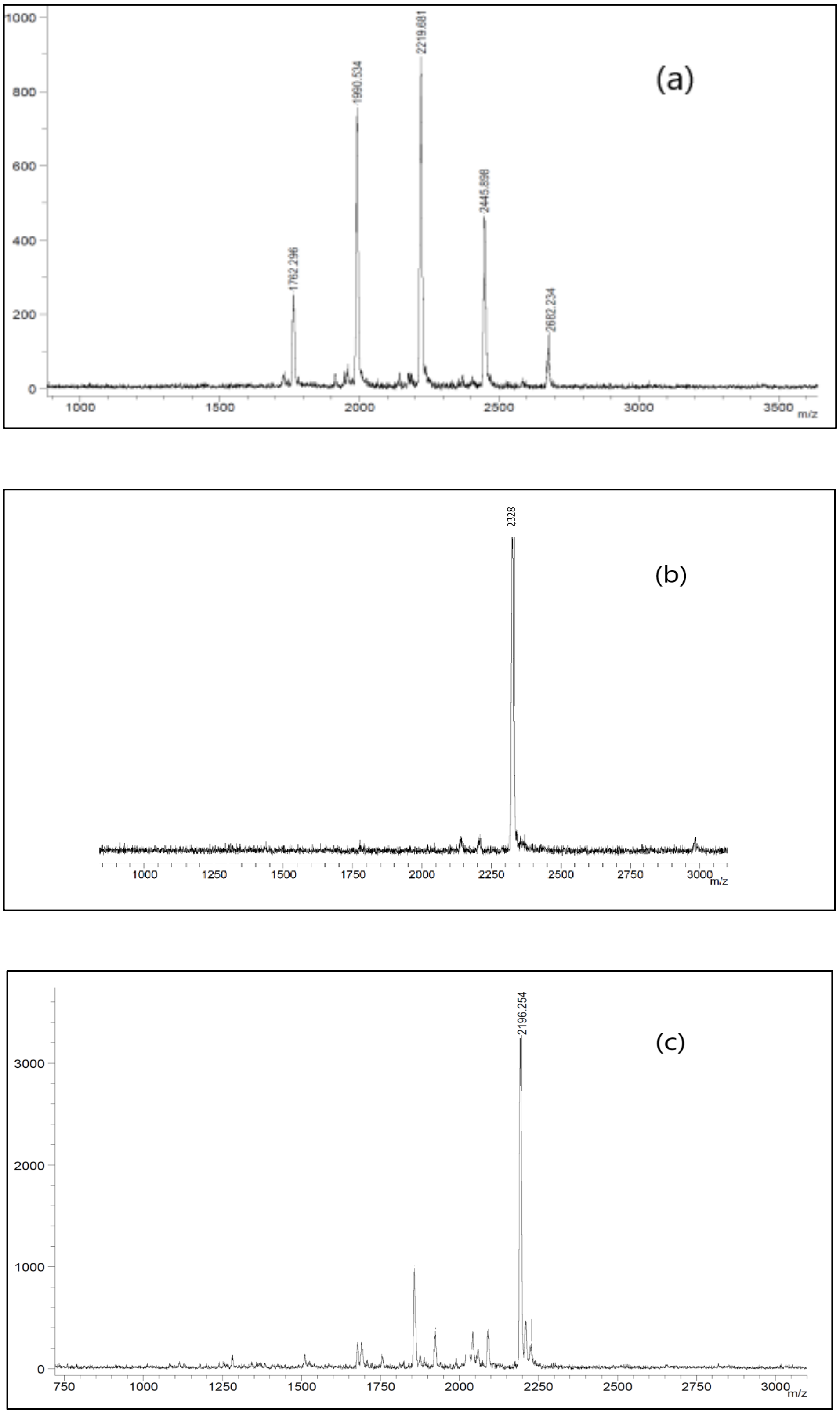

Figure S1. Compounds of mass spectrum of **4** (2682 $[M]^+$) (**a**), **5** (2328 $[M+H]^+$) (**b**), and **6** (2196 $[M+H]^+$) (**c**).

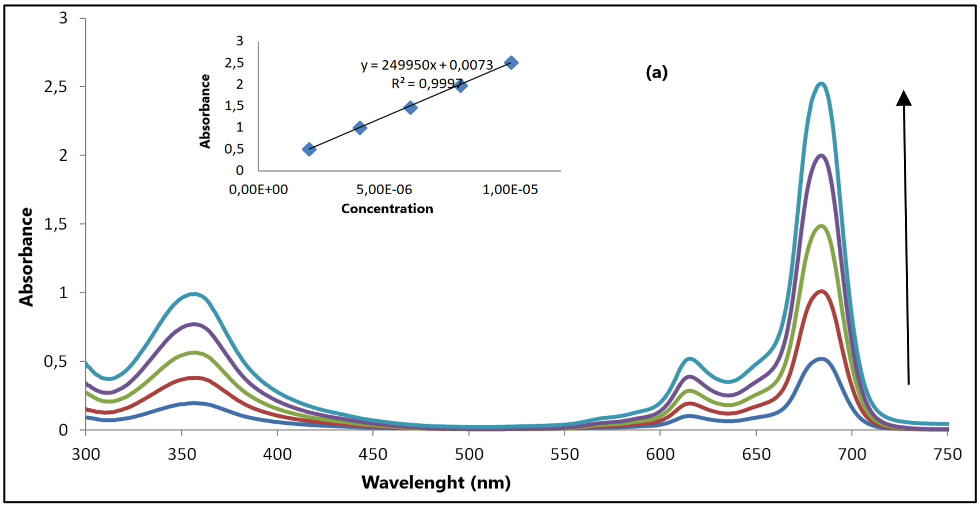

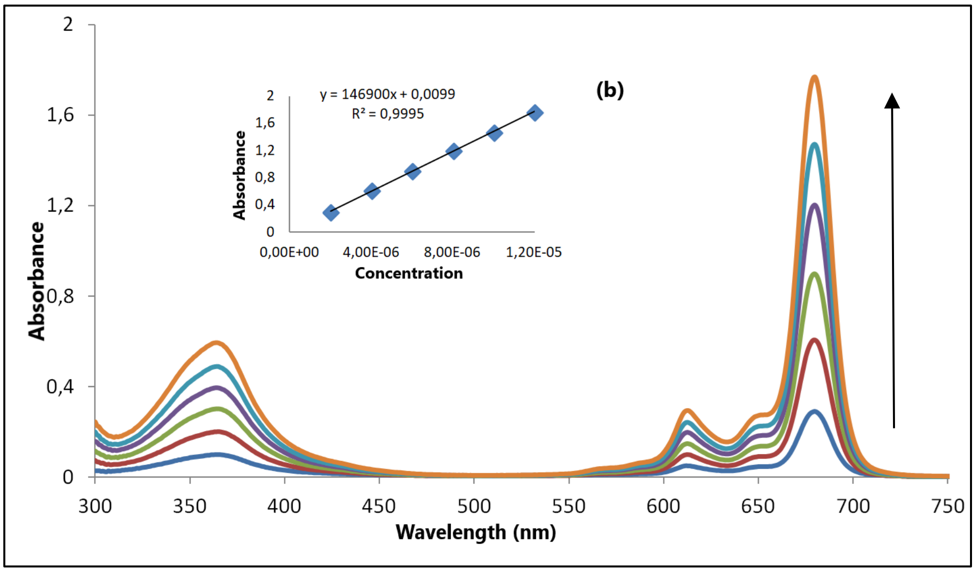

Figure S2. Absorption spectra of **4** **(a)** in DMSO and **5** **(b)** in DMF at different concentration: 2 x 10^{-6}, 4 x 10^{-6}, 6 x 10^{-6}, 8 x 10^{-6}, 10 x 10^{-6} , 12 x 10^{-6} mol dm^{-3}

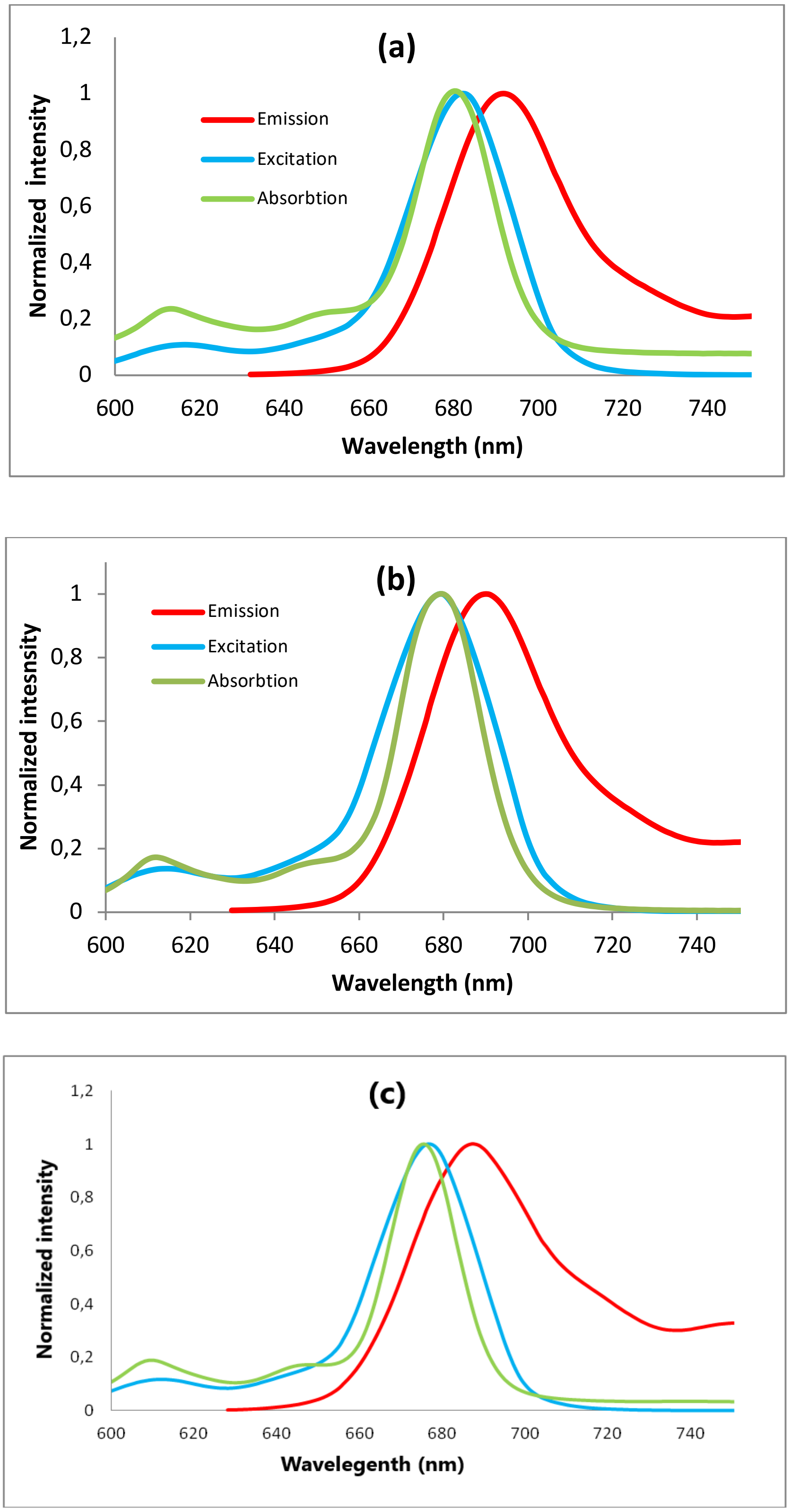

Figure S3. Absorption, excitation and emission spectra of the compounds **4** in DMSO **(a)**, **4** in DMF **(b)**, **6** and in THF **(c)**

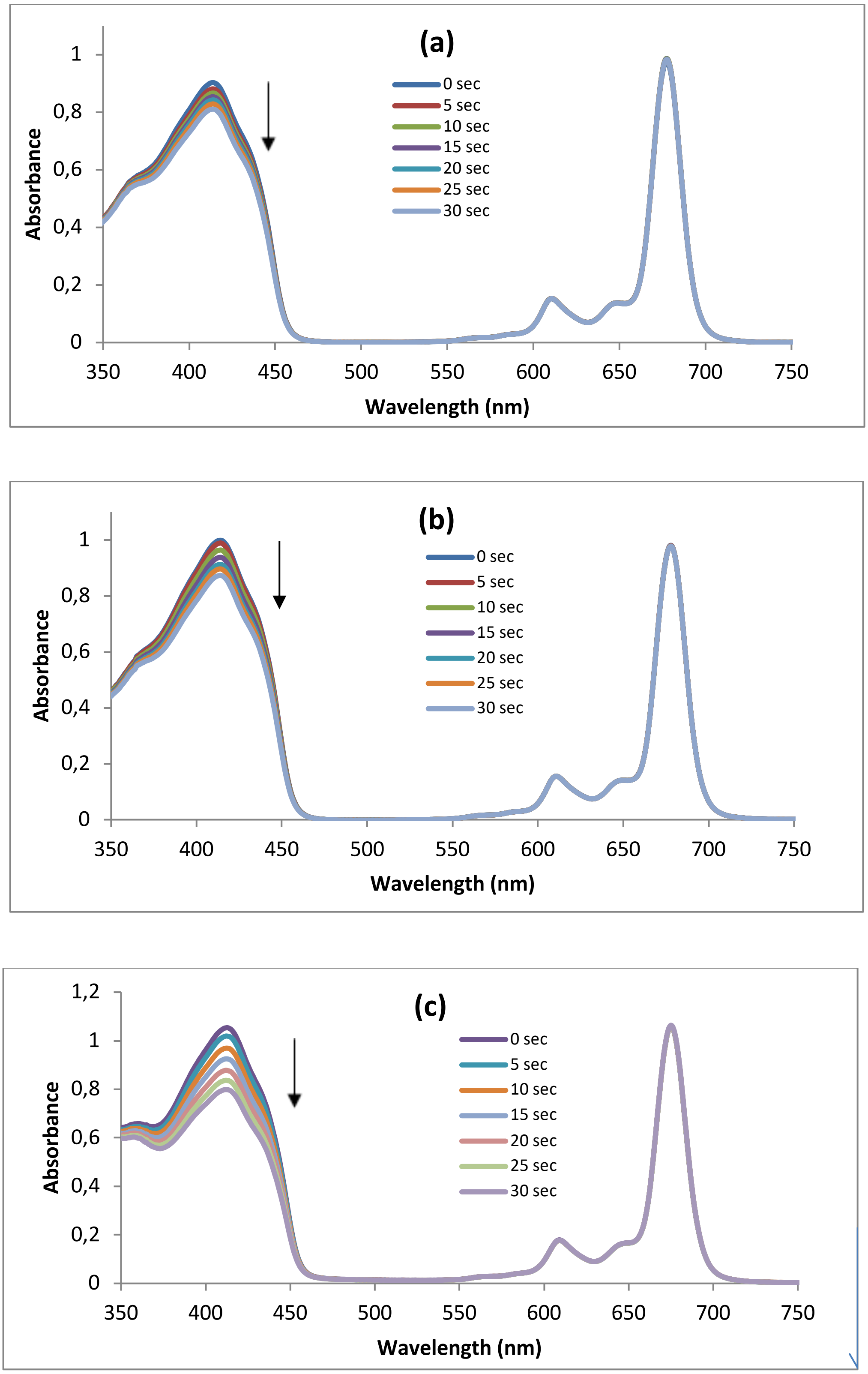

Figure S4. A typical spectrum for the determination of singlet oxygen quantum yield of for complex **5** (**a**) in DMF, **6** (**b**) in DMF and **4** (**c**) in THF at a concentration 6 x 10^{-6} mol dm^{-3}

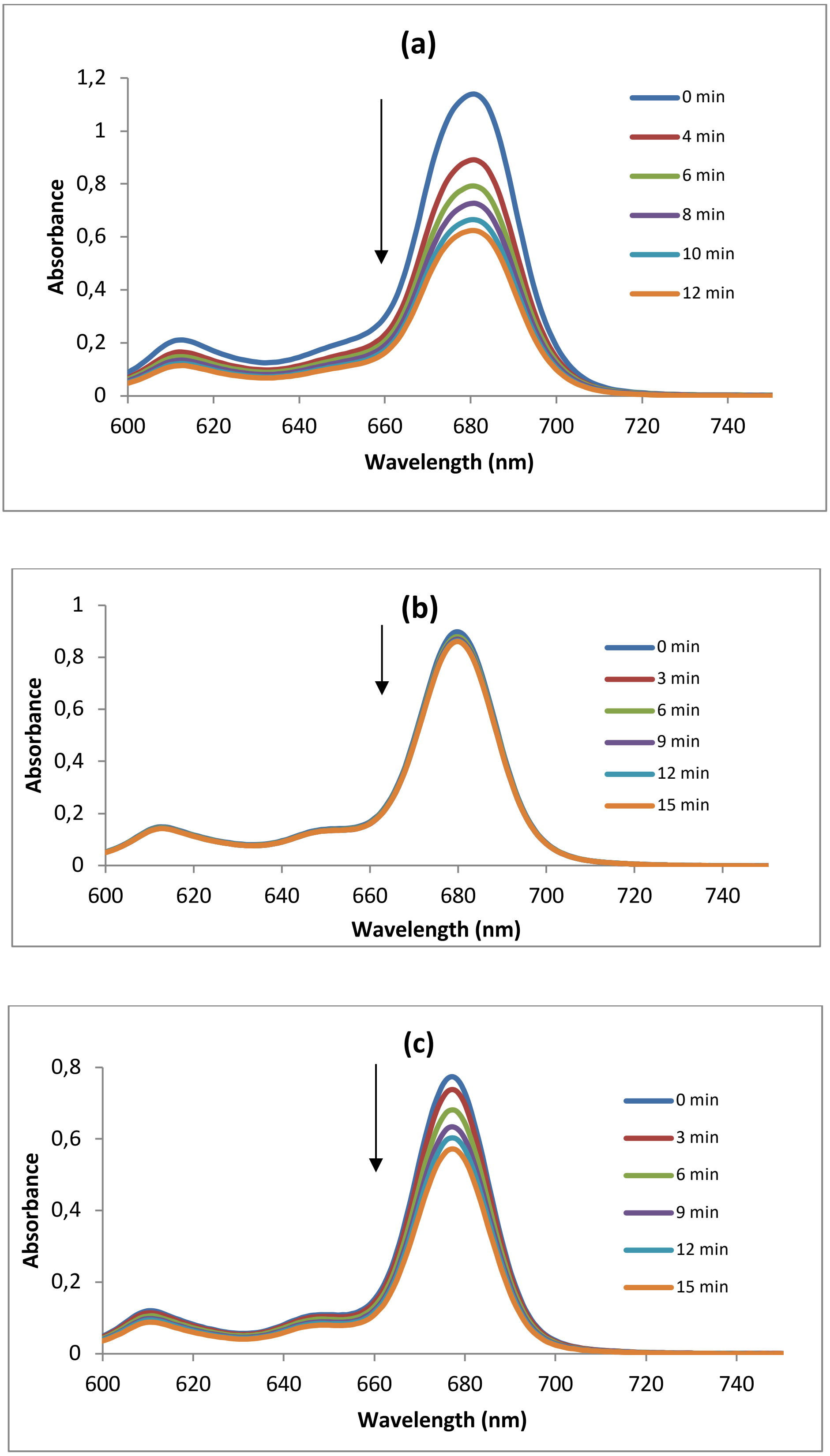

Figure S5. A typical spectrum for the determination of Photodegradation. This figure was for complex **4** in DMF (**a**), **5** in DMSO (**b**) and **5** in DMF (**c**)

References

S1. Frey-Forgues S, Lavabre D. Are fluorescence quantum yields so tricky to measure? A demonstration using familiar stationery products. Journal of Chemical Education 1999; 76: 1260-1264. doi: 10.1021/ed076p1260

S2. Ogunsipe A, Chen JY, Nyokong T., Photophysical and photochemical studies of zinc(II) phthalocyanine derivatives effects of substituents and solvents. New Journal of Chemistry 2004; 25: 822-827. doi: 10.1039/B315319C

S3. Durmus M, Nyokong T. Photophysicochemical and fluorescence quenching studies of benzyloxyphenoxy-substituted zinc phthalocyanines. Spectrochim Acta A. 2008; 69: 1170-1177. doi: 10.1016/j.saa.2007.06.029

S4. Saka ET, Durmus M, Kantekin H. Solvent and central metal effects on the photophysical and photochemical properties of 4-benzyloxybenzoxy substituted phthalocyanines. Journal of Organometallic Chemistry 2011; 696: 913-924. doi: 10.1016/j.jorganchem.2010.10.024

S5. Brannon JH, Madge D. Picosecond laser Photophysics. group 3A phthalocyanines. Journal of the American Chemical Society 1980;102: 62-65

S6. Ogunsipe A, Nyokong T. Photophysical and photochemical studies of sulphonated non-transition metal phthalocyanines in aqueous and non-aqueous media. Journal of Photochemistry and Photobiology A: Chemistry 2005; 173: 211-220. doi: 10.1016/j.jphotochem.2005.03.001

S7. Seotsanyana-Mokhosi I, Kuznetsova N, Nyokong T. Photochemical studies of tetra-2,3-pyridinoporphyrazines. Journal of Photochemistry and Photobiology A: Chemistry 2001; 140: 215-222. doi:10.1016/j.jphotochem.2005.03.001

S8. Kuznetsova N, Gretsova N, Kalmykova E, Makarova E, Dashkevich S, Negrimovskii V, Kaliya O, Luk'yanets E. Structure-photochemical properties relationship for porphyrins and related compounds, Russ. J. Gen. Chem. 2000; 133:133-140.

S9. Spiller W, Kliesch H, Wohrle D, Hackbarth S, Roder B, Schnurpfeil G, Singlet oxygen quantum yields of different photosensitizers in polar solvents and micellar solutions. Journal of Porphyrins and Phthalocyanines 1998; 2: 145-149. doi:10.1002/(SICI)1099-1409(199803/04)2:2<145::AID-JPP60>3.0.CO;2-2

S10. Ogunsipe A, Maree D, Nyokong T. Solvent effects on the photochemical and fluorescence properties of zinc phthalocyanine derivatives. Journal of Molecular Structure 2003; 650: 131-140. doi:10.1016/S0022-2860(03)00155-8
